# Renal Biopsy Research in the Former Soviet Union: Prevention of a Negligent Custom

**DOI:** 10.5402/2013/980859

**Published:** 2012-09-04

**Authors:** Sergei V. Jargin

**Affiliations:** Department of Pathology, Peoples' Friendship University of Russia, Clementovski Per 6-82, Moscow 115184, Russia

## Abstract

Insufficient international coordination of medical research and partial isolation from the international scientific community can result in repetition of research already performed in other countries. Renal biopsy was broadly used for research in the former Soviet Union. It was performed, sometimes without sufficient clinical indications, in patients with amyloidosis, renovascular hypertension (from both kidneys: on the side of the renal artery stenosis and the contralateral one), chronic alcoholism, and acute and chronic pyelonephritis (intraoperative wedge and core biopsies). In chronic alcoholism, biopsies were taken from kidneys, pancreas, salivary glands, stomach, lung, skin, and liver, sometimes repeatedly. The classification of glomerulonephritis was different from those used internationally, for example, it did not include IgA nephropathy as a separate entity. Several examples of studies based on renal biopsies are discussed in this paper. A conclusion is however optimistic: the upturn in economy enables today to modernize equipment and introduce new methods, while broadening international cooperation facilitates the flow of foreign experience into the country. The purpose of this paper was to prevent inadequate use of renal biopsy in future.

Insufficient international coordination of medical research and partial isolation from the scientific community can result in unnecessary experiments and clinical studies with repetition of research already performed in other countries; while contraindications, known from the literature, are sometimes disregarded. Renal biopsy (RB) was broadly used for research in some institutions of the former Soviet Union (SU), for example, an overview encompassing the years 1970–1999 analyzed 4400 core biopsies from kidneys taken at the Moscow Medical Academy [1]. Even if RB is performed according to the indications, a part of the specimen is sometimes consumed for purely scientific purposes, which can be justified if research is bona fide. Electron microscopy was rarely used for the diagnostic purposes; nevertheless, 1/3 of the renal tissue cylinder was taken for resin embedding. Semithin (around 4 *μ*m) epoxy resin sections, often providing significant information, were made in many cases for scientific purposes but were usually not used for diagnostics, the latter being performed mainly on the basis of paraffin sections and immunofluorescence. The number of RB performed yearly has decreased since the 1980s; however, today there are more funds for research, and it might be useful to recollect some experience from the recent past, to ensure more responsible attitude in future. RB were taken for research from patients with amyloidosis [2–5], renovascular hypertension (from both kidneys: on the side of the renal artery stenosis and the contralateral one) [6–11], chronic alcoholism [12–14], and acute and chronic pyelonephritis (intraoperative wedge and core biopsies) [15–18], sometimes without clear clinical indications. Note that pyelonephritis is generally not listed in handbooks among conditions where RB is indicated; while acute inflammation (pyonephrosis) is regarded as a contraindication to the RB [19]. Use of RB (fine-needle) in acute pyelonephritis was reported in a recent scientific publication [20]. Apart from the above-referenced papers, no other studies based on RB in acute pyelonephrities are known to us, while in chronic pyelonephritis no other large-scale studies performed abroad later than in the 1960s [21] have been found. Furthermore, the concept of informed consent has not been sufficiently known and observed in the former SU. Identification of the amyloid type was not performed, which could have lowered clinical value of the biopsies; while RBs were sometimes taken also in those cases when amyloidosis could have been confirmed by rectal or gingival biopsy, in familial Mediterranean fever or secondary amyloidosis. In chronic alcoholism, biopsies were taken from kidneys, pancreas, salivary glands, stomach, lung, skin, and liver, sometimes repeatedly [14]. It should be commented that alcoholics and people with alcohol-related dementia have been a vulnerable population group in the former Soviet Union since the mid-1980s at least; and offences against them have often been tolerated by the society and its institutions. In the author's opinion, RBs have sometimes been performed without sufficient indications also in glomerulonephritis (Gn); which is less obvious because RB was regarded to be indicated if Gn had been suspected. Indications for the RB are not discussed here; it should be mentioned however that the classification of Gn applied in the SU was different from those used internationally. For example, the domestic classification did not include IgA nephropathy as a separate entity, although this condition has therapeutic approaches of its own [22]. IgA nephropathy was often diagnosed on biopsy as mesangioproliferative Gn; the latter designation being broadly used, comprising over 50% of all Gn cases diagnosed on biopsies [1], often without further definition, which must have lowered clinical value of RB. IgA-nephropathy was not mentioned even in the article from a leading institution dedicated to the “hematuric form” of chronic Gn [23]. At the same time, special variants of Gn, absent in the international literature, were proposed [24–27]. Methods of glomerular morphometry, used earlier in [4, 10, 28, 29] and criticized in [11], as well as similar ones, were applied by other scientists [26, 27] without references. Furthermore, research already performed abroad was occasionally repeated in some form in the SU because of the partial isolation from the international scientific community and insufficient availability of foreign professional literature [30, 31].

From 1982 to 1990, I was a Trainee and then Practical Pathologist and Lecturer at the Department of Pathological Anatomy of the Moscow I.M. Sechenov Medical Academy, attended meetings of Moscow Society of Pathology, and heard many scientific reports. The best studies in pathology followed the following pattern: a large amount of foreign literature was worked over and a meaningful concept formulated. Specimen were processed using modern methods. Resulting publications contributed to dissemination of modern scientific knowledge. It should be noted that even in such cases the principle of priority was sometimes violated if results published by foreign authors were directly or indirectly presented as own achievements [31, 32]. Along with publications having value of review with testing and adaptation of foreign results to domestic conditions, numerous useless and partly fabricated reports were published. Unreliable publications originated also from renowned institutions (discussed in [32–35]). However, there was also serious research in some fields of medical science. With regard to the implications for practice, it is difficult to assess the extent of harm, having only a limited field of view and no statistical data. In connection with the topic of this paper, some risk for patients could have been caused, for example, by the core biopsies from contralateral (to the renal artery stenosis) kidneys in renovascular hypertension, taken for research. English summaries of some articles, authored by scientists from a leading institution in Moscow, provide some insight with regard to research quality: “Comparison of the findings of clinical, instrumental, and laboratory examination of patients with vasorenal [i.e., renovascular] hypertension with the results of morphological analysis of renal bioptic material showed that multivariate regression analysis of the parameters of examination of the patients provides for authentic calculation of the quantitative index of nephroarteriolosclerosis—the vascular index of the afferent arterioles of the renal glomeruli. The calculated values of the vascular index for both kidneys are criteria for choosing the method of operative intervention in vasorenal hypertension,” that is, reconstructive vascular operations versus nephrectomy [36]. At that time I practiced at the same department and participated in some studies on renovascular hypertension [10, 37–39]. Most of the biopsy specimens from contralateral kidneys were small, contained 1–3 glomeruli and arterioles, or none of them at all. A majority of the specimen were unsuitable for reliable morphometry. I discussed it with the head researcher and other participants of the study, but they insisted on continuation. In 1990 I refused to take part in a next study, having said to the head that I would prefer to avoid participation in scientific misconduct. From 1990 to 1995 I practiced pathology abroad.

Another summary [9]: “The renin-angiotensin (juxtaglomerular apparatus—JGA) and prostaglandin [interstitial cells (IC) of renal medulla and nephrocytes of collecting tubules (NCT)] systems of the kidneys were studied in 72 patients (renal biopsies, nephrectomy, morphofunctional correlations) with the nephrogenic arterial hypertension (vasorenal hypertension, chronic glomerulonephritis, pyelonephritis). Histologic and electron-microscopic methods were used; the renin activity was determined in the peripheral blood and blood from the renal veins. The results were analyzed mathematically and statistically using an original program. It is shown that stereotype cyclic changes develop in the endocrine renal system of patients with renal hypertension and that they reflect the stages of initial hyperfunction (ultrastructural hyperplasia of JGA cells with appearance of numerous immature granules; ultrastructural moderate hyperplasia of medulla IC; increase of blood renin activity), discoordination of functions (progressing JGA hyperfunction and depletion of prostaglandin synthetic function of medulla IC; compensatory activation of NCT; further increase of the blood renin activity) and depletion (atrophy and fibroblastic transformation of the JGA of the majority of nephrons and of medulla IC). The stages of renal endocrine system alterations in the arterial hypertension are the manifestation of compensatory and adaptive response. Morphofunctional analysis with the use of morphometry and mathematical statistics are necessary for the objective evaluation of this response” [9].

Quality of documentation of this concept is discussed and illustrated in [33]. Late in the 1980s, I searched through the archive of ultrastructural images and photographic plates and found about 20–30 photographs of JGA cells with numerous secretory granules and rhomboid protogranules. Some images had similar morphology and probably originated from a limited number of patients and experimental animals. These photographs were used as illustrations in dissertations, numerous articles, and monographs. There was not enough material for reliable statistical assessment of the relative volume of secretory granules and other ultrastructural morphometric indices. Images of human renomedullary IC, bona fide suitable for the quantitative evaluation of prostaglandin synthesis, were not found in the archive. There were only a few ultrastructural images of IC, shown in [33], which were repeatedly used as illustrations. The phenomenon referred to in the above summary as “compensatory activation of nephrocytes of collecting tubules (NCT)” [9], as a proposed morphologic equivalent of enhanced synthesis of antihypertensive factors, has never been clearly illustrated. The data about “stereotype cyclic changes in the endocrine renal system” [9] in glomerulonephritis, pyelonephritis, and other renal diseases have never been confirmed by other researchers. Representative sets of electronograms, required for statistical analysis of morphometric data, have never existed. I observed how the morphometry was performed and sometimes participated. It was done by laboratory personnel on ultrastructural photographs about 10 cm in size, by means of a ball-point pen connected to an image analyzer. Only the granule-containing cells were processed morphometrically; the mean level of granularity was not determined even for a single JGA, let alone representative assessment of different JGA in order to characterize the whole kidney. Rhomboid protogranules and spherical secretory granules were evaluated together with nonspecific lipofuscin-like granules, which is known as a potential source of confusion when the degree of granularity is estimated [40].

The last quotation: “Light-optical, electron microscopic and morphometric studies of biopsy material from kidneys of 24 patients with vasorenal hypertension were performed. It was determined that sclerosis of the arteries and arterioles on the side of the lesion was more marked, than in the contralateral kidney. At a later stage of the disease (more than 3 yr.) there were occasional signs of increased renin-synthesizing activity of the juxtaglomerular apparatus (JGA) with its simultaneous decrease at the stenotic side. JGA activation in the contralateral kidney can be one of the mechanisms supporting hyperrenin arterial hypertension and can prevent the decrease of the blood pressure after surgery. Studying of the biopsy material with the estimation of the sclerosis degree of the arterial tree vessels, as well as JGA activity, is important for prognosing the results of surgical treatment of vasorenal hypertension.” [10]. It should be commented that the morphometric indices characterizing arteriolosclerosis (so-called vascular indices) were fabricated or trimmed by myself, in order to obtain desirable results. Unsuitability of RB specimens for reliable morphometry was discussed with the coauthors. Questionable reliability of JGA activity assessment was discussed above. Therefore, practical recommendations with regard to the treatment of renovascular hypertension, formulated in this and in other publications by the same research group, are, in my opinion, unreliable. Numerous articles, some of them with practical recommendations, were published on the basis of this research series [9, 10, 36–39, 41–48]; some of them were criticized in [11, 32, 33]. Moreover, I fabricated morphometric data in [4, 28, 29, 49], which was described in more detail in the Russian language publication [11]. Apart from [11], messages of retraction sent to the corresponding journals, have not been published.

Information provided by this paper can be of interest because of the broadening international cooperation in research [34]. Today, however, there are grounds for optimism. Russian medical science is standing on the eve of great changes: the upturn in economy enables to modernize equipment and introduce new methods, while broadening international cooperation facilitates the flow of foreign experience into the country. Hopefully, the international cooperation will contribute to elimination of the drawbacks discussed above. In conclusion, this paper has been submitted to the open access Journal to prevent inadequate use of RB in future. Performing RB, it should be always kept in mind that there must be a favorable risk-benefit ratio for the patient.
